# *Neobalantidium coli*: First molecular identification from the Eurasian wild boar, *Sus Scrofa* in Bushehr Province, Southwestern Iran

**DOI:** 10.1002/vms3.210

**Published:** 2019-10-24

**Authors:** Shamsi Noorpisheh Ghadimi, Mohammad R. Abedini, Bahador Sarkari, Amir Savardashtaki, Fattaneh Mikaeili

**Affiliations:** ^1^ Department of Parasitology and Mycology School of Medicine Shiraz University of Medical Sciences Shiraz Iran; ^2^ Department of Parasitology and Mycology Fasa University of Medical Sciences Fasa Iran; ^3^ Basic Sciences in Infectious Diseases Research Center Shiraz University of Medical Sciences Shiraz Iran; ^4^ Department of Medical Biotechnology School of Advanced Medical Sciences and Technologies Shiraz University of Medical Sciences Shiraz Iran

**Keywords:** genetic diversity, genotype, Iran, *Neobalantidium*, *Sus scrofa*, wild boars

## Abstract

*Balantidium coli* is a common parasite of pig and wild boars (*Sus scrofa*) which can infect humans and several species of mammals. This study aimed to determine the genotype of *Balantidium* isolated from Eurasian wild boars in Bushehr province, Southwestern Iran. Twenty‐five faecal samples, originating from 25 wild boars captivated in our previous study, were processed. DNA was extracted from the faecal samples and PCR‐amplified, targeting an ITS1–5.8s‐rRNA–ITS2 region of *Balantidium* genome. PCR product was purified from the gel, and sequenced. BLAST analysis was performed in order to compare our isolates with other previously reported ones. A phylogenetic tree was constructed, using MegaX software, to find out the phylogenetic diversity of the isolates. With PCR it was possible to detect *Balantidium* DNA in the faecal samples of 13 out of 25 (52%) of the wild boars. BLAST analysis of seven isolates revealed that the isolates belong to the newly introduced genus *Neobalantidium coli*. Sequences of three isolates were deposited in the GenBank. Moreover, molecular analysis revealed six areas of nucleotide differences within the isolates and nine areas of difference between the sequences obtained in this study and those available in the GenBank. Phylogenetic analysis revealed that the sequences of isolates of this study have up to 2.2% dissimilarity from those published in the GenBank. The findings of this study, for the first time, revealed that some of the isolates of *Balantidium* originating from wild boars in Southwestern Iran belonged to the *N. coli.*

## Graphical abstract

Wild boars (*Sus scrofa*) are considered as the reservoirs of human balantidiasis in Iran. Recently, based on genetic analysis of *Balantidium* isolated from different hosts a new genus, *Neobalantidium* has been proposed. Findings of the current study, for the first time, revealed that some of the isolates of *Balantidium* originating from wild boars in Southwestern Iran belong to the newly introduced genus, *Neobalantidium coli*.

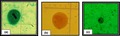

## 1 INTRODUCTION

*Balantidium coli* is a ciliated protozoan that lives in the large intestine (caecum and colon) of pigs (the natural reservoir host), human, nonhuman primates and rodents. *Balantidium coli* is the largest and only ciliate protozoan that infects humans (Schuster & Ramirez‐Avila, 2008). Humans get infected accidentally through eating of water and food contaminated with the parasite cyst (Schuster & Ramirez‐Avila, 2008). Balantidiasis is usually sporadic in humans, and temporary contamination of water can raise human infection in some areas. *Balantidium coli* is often a neglected pathogen and recently is considered as an emerging and re‐emerging protozoan. In human, the disease might be asymptomatic or may have a range of presentations including; nonbloody diarrhoea, cramping, halitosis, abdominal pain and sometimes mucoid bloody diarrhoea. The vast majorities of infections are asymptomatic or may just cause moderate diarrhoea (Schuster & Ramirez‐Avila, 2008). With a global prevalence of 0.02%–1%, balantidiasis is a significant problem in some of the developed countries with a high prevalence rate in Latin America, the Philippines, Papua New Guinea, Western New Guinea and Middle East (Esteban, Aguirre, Angles, Ash, & Mas‐Coma, 1998; Maleky, 1998; Schuster & Ramirez‐Avila, 2008). Pigs are the main reservoir of *B. coli* and the percentage of infection in the diverse populations of pigs reported to be 20%–100% (Schuster & Ramirez‐Avila, 2008; Solaymani‐Mohammadi et al., 2004; Yaghoobi, Sarkari, Mansouri, & Motazedian, 2016). Along with domestic pigs, wild boars (*Sus scrofa*) are considered as the reservoirs of *Balantidium* infection in human (Cox, 2005; Nakauchi, 1999; Solaymani‐Mohammadi, Rezaian, & Anwar, 2005). *Sus scrofa* is the most widespread species of wild pig, distributed from Western Europe to the Far East and Southeast. Wild boars can be found in various regions of Iran, especially in the north‐northwest, southwest and southeast forests. Several subspecies of wild boars have been reported in Iran, although their exact boundaries are not clear. These subspecies are including *S. s. attila*, *S. s. ni‐gripes*, *S. s. davidi* and *S. s. lybicus*. The *S. scrofa* or the Eurasian Wild Pig is the most common species of wild pigs. *S. s. davidi* considered being the subspecies in central and presumably southern and eastern Iran (Available at https://www.researchgate.net/publication/235967637_Sus_scrofa_subspecies_of_Iran). They may act as the reservoir of several parasitic infections, including balantidiasis (Sarkari, Mansouri, Khabisi, & Mowlavi, 2015; Yaghoobi et al., 2016; Mansouri, Sarkari, & Mowlavi, 2016; Sarkari et al., 2018).

*Balantidium coli* is a common protozoan infection in wild boars in Iran with prevalence rates of 25%–70% (Solaymani‐Mohammadi et al., 2005, 2004; Yaghoobi et al., 2016). Solaymani‐Mohammadi et al. reported that 67% of the Eurasian wild boars, *S. scrofa*, in Lorestan Province, western Iran, were infected with *B. coli* (Solaymani‐Mohammadi et al., 2004). Human infection has been reported from Khuzestan and Hormozgan province in the south of Iran (Solaymani‐Mohammadi et al., 2005). Swine balantidiasis is often asymptomatic, yet these asymptomatic pigs serving as the major reservoir hosts of the parasite. Wild boars in rural Western Iran are considered as a reservoir for human balantidiasis.

Due to the pleomorphism of *B. coli* and also the range of its hosts, the *Balantidium* taxonomy is rather controversial (Pomajbikova et al., 2013). A previous study advised that morphological features and host species are insufficient for the identification of *Balantidium* species (Ponce‐Gordo, Jimenez‐Ruiz, & Martinez‐Diaz, 2008). Considering the morphological differences or the host species, a number of *Balantidium* species have been described from various wild and domestic mammals. It has been proposed that *B. coli* be transferred to a new genus, *Balantioides* (Mathison & Pritt, 2019). Recently, based on genetic analysis of *Balantidium* isolated from different hosts, Pomajbíková et al. (2013) proposed a new genus, *Neobalantidium*, to accommodate *B. coli* and other *Balantidium* species of warm‐blooded hosts (Pomajbikova et al., 2013). *Neobalantidium* is considered to be a junior synonym of *Balantioides* (Chistyakova, Kostygov, Kornilova, & Yurchenko, 2014; Pomajbikova et al., 2013). As *B. coli* lack mitochondria, the only available genetic data for *B. coli* are from the nuclear small subunit rRNA gene (SSU‐rDNA) and the internal transcribed spacer (ITS1‐5.8S rDNAITS2) regions. For differentiating among narrowly related subtypes, analysis of the ITS region, and particularly the ITS2 fragment, is considered the best possibility.

Given the worldwide distribution of *Balantidium* in different hosts and its unknown epidemiology in human populations, the need for further studies on the genetic diversity of this pathogen is evident (Pomajbikova et al., 2013). So far, there has been no molecular study to identify the genotypes of *Balantidium* in domestic or wild boars in Iran. Therefore, this study, for the first time, aimed to determine the genotypes of *Balantidium* isolated from wild boars in the southwest of Iran.

## 2 MATERIALS AND METHODS

### 2.1 Ethics

The study was approved by and carried out under the guidelines of the Ethical Committee of the Shiraz University of Medical Sciences.

### 2.2 Study area

Bushehr province is located at 28.9184° N; 50.8382° E and Dilam Port (where the samples were collected) is located at the north of the province. The climate of the Dilam Port is warm and humid and is mainly covered with massive forests and pasture plants.

### 2.3 Sample collection

In this study, stool samples collected from the gastrointestinal tracts of 25 wild boars in our previous study, from the northern regions of Bushehr Province were evaluated (Mansouri et al., 2016).

### 2.4 Microscopical identification of *Balantidium*

Temporary staining of stool samples with Lugol's solution was performed. Moreover, stool smears were prepared and stained with both trichrome and Ziehl–Neelsen stains (Yaghoobi et al., 2016).

### 2.5 DNA extraction and PCR

Total genomic DNA was extracted from the wild boars' faecal samples, using the Nucleic Acid Extraction Kits (Vivantis), following the manufacturer’ instructions. The ITS1–5.8s‐rRNA–ITS2 region of *Balantidium* was amplified, using the forward B5D (5‐GCTCCTACCGATACCGGGT) and the reverse B5R (5‐ATATGCTTAAGTTCAGCGGG) primers (Ponce‐Gordo et al., 2008). PCR was carried out in a 25 μl reaction volume, containing 2 μl of template DNA (50 ng), 0.5 μl of each primer (10 pmol/μL), 12.5 μl of master mix (Qiagen) and 9.5 μl of ddH_2_O. PCR was performed with the following strict temperature profile; initial denaturation of 94°C, for 5 min followed by 30 cycles of 94°C, 60°C and 72°C, each for 1 min and final extension at 72°C for 5 min. *Balantidium* DNA, extracted from a parasitologically confirmed case, as positive control, and ddH_2_O as negative controls were included in each run of the experiment. The resulting products were separated by gel electrophoresis on a 1.5% agarose gel, stained with GelRed (GelRed^®^, Biotium) for visualization under a BioDoc gel documentation System (UVP). PCR products were purified from the gel, using a Gel Extraction Kit (Vivantis), according to manufacturer's instructions and sequenced in both directions, using the same primers employed in the PCR. Sequence results were analysed by the Geneious software (http://www.geneious.com) and compared with sequences available in GenBank, using the BLAST system (http://www.ncbi.nlm.nih.gov/). The phylogenetic relationships were constructed using sequences obtained in this study and reference sequences deposited in GenBank, using the maximum‐likelihood method, based on the Kimura two‐parameter model, using Mega‐X software. Bootstrap analyses (using 1,000 replicates) were carried out to determine the robustness of the finding.

## 3 RESULTS

**Figure 1:**
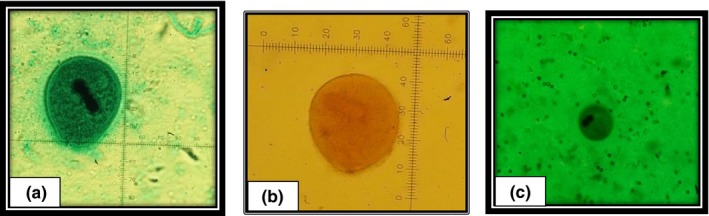
Trophozoites and cyst of *Neobalantidium coli* isolated from wild boars faecal samples. (a) Trophozoite of *N. coli* stained with Trichrome; (b) Trophozoite of *N. coli* stained with Lugol's iodine; (c) Cyst of *N. coli*, stained with Trichrome (Mag. 40 X)

**Figure 2:**
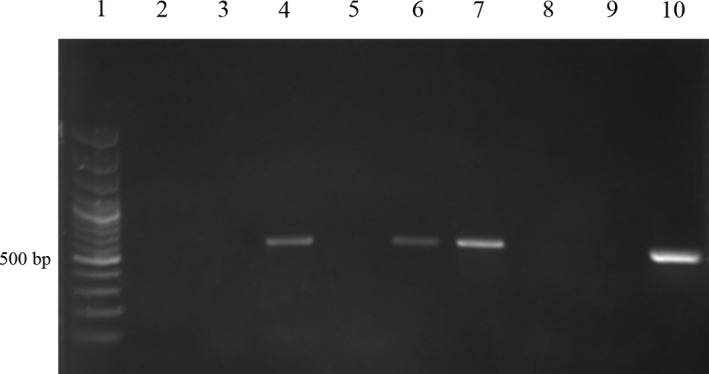
Electrophoresis of ITS1‐5.8s‐ITS2 region of *Neobalantidium coli* in samples of wild boars in Bushehr province, southern Iran. Lane 1: molecular marker, lane 4, 6, 7 and 10: positive samples; lane 2, 3, 5, 8 and 9: negative samples

**Figure 3:**
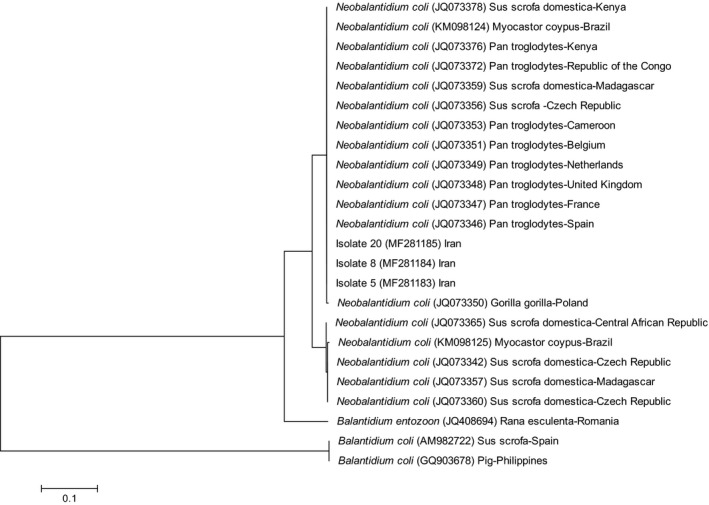
Phylogenetic tree (using maximum likelihood method) of ITS1‐5.8s ‐ITS2 region of *Neobalantidium coli* isolates obtained in this study and references sequences retrieved from GenBank

Trophozoites or cysts of *Balantidium* were detected in 16 out of 25 (64%) of stool samples of wild boars by microscopy (Figure 1). The PCR result revealed an approximate 550 bp band of *Balantidium* in 13 out of 25 (52%) faecal samples (Figure 2). The sequence analysis was performed for three isolates to characterize the samples and BLAST system revealed that the isolates in this study had the highest degree of similarity with the newly introduced genus of *Balantidium* known as *Neobalantidium coli*. The consensus sequences determined in this study were deposited in the GenBank database under the accession numbers MF281183 to MF281185. The phylogenetic tree indicated that three isolates of *N. coli* obtained in this study were taxonomically grouped into one clade and one haplotype (Figure 3). The ITS1‐5.8s‐ITS2 sequence of isolate 5 (Accession number MF281183), isolate 8 (Accession number MF281184) and isolate 20 (Accession number MF281185) of *N. coli* obtained in this study had 100% homology with each other. Based on the pairwise comparison, intra‐species genetic diversity within isolates of *N. coli* amounted to 0%–6%.

## 4 DISCUSSION

*Balantidium* is a common parasite among pigs that can infect humans and many other species of mammals (Schuster & Ramirez‐Avila, 2008). Balantidiasis is considered as an often‐neglected disease which has not received much attention. Human reported cases have been associated with contact with domesticated pigs. However, there is still controversy about the epidemiology of *Balantidium* infection in humans. Several cases of *Balantidium* infection have been reported in humans who have no contact with pigs, such as those reported in Muslim countries (Maleky, 1998). In contrast, residents of endemic areas with a high prevalence of infection in pigs are often reported as having negative or an asymptomatic *Balantidium* infection (Schuster & Ramirez‐Avila, 2008). Cases of human balantidiasis have been previously reported from the Persian Gulf region in Iran (Maleky, 1998). Considering the fact that raising and breeding of domestic pigs are banned in Iran due to Islamic law, it can be assumed that wild boars are involved in the transmission of *B. coli* to humans in this area. In view of that, wild boars may be considered as the leading reservoir of *B. coli* in the region.

Molecular studies of *B. coli* by Ponce‐Gordo (2008) revealed two clearly different genotypes in the ITS1 and ITS2 regions of the pig isolates (Ponce‐Gordo et al., 2008). Using two gene markers, SSrDNA and ITS1‐5.8SDNA‐ITS2, and considering the genetic diversity of *B. coli* Pomajbíková (2013) introduced the new *Neobalantidium* genus to house *B. coli* and other *Balantidium* species of warm‐blooded hosts (Pomajbikova et al., 2013). In this study, the genetic diversity of *B. coli* isolated from the wild pigs of the Bushehr province, together with reference sequences of *B. coli* ciliates was analysed, using ITS1‐5.8SrRNA‐ITS2 as a phylogenetic marker. Findings of our study revealed that the isolates of *Balantidium,* originating from wild boars from southwest of Iran, belongs to the genus *N. coli*.

So far, genetically characterized *N. coli* has been reported from the brain of a coypu, *Myocastor coypus* from Brazil (GenBank Accession no.: KM098125.1), from *Homo sapiens* from Argentina (GenBank Accession no. KR349515.1–KR349517.1), from *S. scrofa domestica* from Brazil (GenBank Accession no. KR349500.1–KR349514.1), from *S. scrofa* domestica from Cameron, Kenya, Czech Republic, Central African Republic and Spain; from *Pan troglodytes* in Kenya, Republic of the Congo, Netherland and Cameron and from *Gorilla gorilla* from Poland (Hassell et al., 2013; Oliveira Costa et al., 2013; Pomajbikova et al., 2013; Ponce‐Gordo et al., 2008).

The ITS1‐5.8s‐ITS2 sequence of the isolates in this study had 100% homology with *N. coli* isolated from rodent in Brazil (Accession no. KM098124), from domestic pig in Kenya (Accession no. JQ073378) and in Madagascar (Accession no. JQ073359), from wild boar in Czech Republic (Accession no. JQ73356) and from chimpanzee in Kenya (Accession no. JQ073376), in Spain (Accession no. JQ073346), in France (Accession no. JQ073347), in United Kingdom (Accession no. JQ073348), in Republic of the Congo (Accession no. JQ073372), in Netherlands (Accession no. JQ073349), in Belgium (Accession no. JQ073351) and in Cameroon (Accession no. JQ073353).

The results obtained from the sequencing of our isolates are similar with the results of studies by Pomajbikova et al. on *N. coli* from the chimpanzee from Congo, Cameroon, the Netherlands, Spain and the wild boars from the Czech Republic and are somewhat dissimilar, in seven nucleotides, to the isolates reported from nutria (*Myocastor coypus*) (Oliveira Costa et al., 2013; Pomajbikova et al., 2013). Ponce‐Gordoa and others demonstrated that the polymorphism in the ITS1 and ITS2 regions of *B. coli* isolates were present within one single species (Ponce‐Gordo, Fonseca‐Salamanca, & Martinez‐Diaz, 2011). Our findings are consistent with Ponce‐Gordoa et al, observations.

In a study on wild boars in Lorestan province, western Iran, 25% of wild boars were found to be infected with *B. coli* (Solaymani‐Mohammadi et al., 2004). These animals were considered as the reservoir of human balantidiasis in this area of the country. In this study, only three isolates were sequenced, and this can be considered as the main drawback of this study.

To date, no studies on molecular analysis of *B. coli* isolated in Iran have been reported and this is the first study which looked at the molecular features of *Balantidium* isolated from wild boars in Iran.

## 5 CONCLUSION

Overall, the findings of this study revealed that some of the isolates of *Balantidium* originating from wild boars from the Southwest of Iran belonged to the newly introduced genus known as *N. coli.* Further studies in this field, especially in other areas of Iran, may explore other aspects of this protozoan in the country.

## CONFLICT OF INTEREST

The authors declare that they have no competing interests.

## ETHICAL STATEMENT

The study was approved by and carried out under the guidelines of the Ethical Committee of Shiraz University of Medical Sciences (SUMS).
